# Supplementary material for: Attributes and factors associated with long covid in patients hospitalized for acute COVID-19: A retrospective cohort study
Source: PLoS One. 2025 Jan 16;20(1):e0317512. doi: 10.1371/journal.pone.0317512 (PMC11737794; doi:10.1371/journal.pone.0317512)
Supplement: S1 File — (DOCX) [file pone.0317512.s001.docx]

# Supplementary tables

**Table S1-1. Comorbidities of the patients**

|  | Frequency (n) | Percentage (%) |
| --- | --- | --- |
| Hypertension | 83 | 33.6 |
| Diabetes | 84 | 34 |
| Dyslipidemia | 25 | 10.1 |
| Asthma | 13 | 5.3 |
| COPD | 3 | 1.2 |
| HIV | 5 | 2 |
| Cerebrovascular disease | 5 | 2 |
| Chronic kidney disease | 12 | 4.9 |
| Cardiac illness | 17 | 6.9 |
| Thyroid disorders | 7 | 2.8 |
| Others | 29 | 11.7 |

**Table S1-2. Complications during admission**

|  | Frequency (n) | Percentage (%) |
| --- | --- | --- |
| ARDS | 18 | 7.3 |
| Secondary bacterial infections | 97 | 39.3 |
| Cardiovascular complications | 2 | 0.8 |
| Thromboembolic complications | 17 | 6.9 |
| Neurologic complications | 1 | 0.4 |
| Others | 17 | 6.9 |

**Table S1-3. Laboratory abnormalities during follow-up**

| Laboratory finding | Frequency (n) | Percentage (%) |
| --- | --- | --- |
| Lymphopenia | 44 | 17.8 |
| Leukocytosis | 36 | 14.6 |
| Raised ESR | 28 | 11.3 |
| Raised CRP | 21 | 8.5 |
| Anemia | 20 | 8.1 |
| Raised creatinine | 14 | 5.7 |
| Raised aminotransferase levels | 9 | 3.6 |
| Raised LDH | 6 | 2.4 |

**Table S1-4. Categorization of chest CT findings in COVID-19 infected patients**

| Chest CT findings* | **Typical appearance** (Peripheral, bilateral, GGO; multifocal GGO of rounded morphology, reverse halo sign or other signs of organizing pneumonia) |
| --- | --- |
|  | **Indeterminate appearance** (Absence of typical features AND presence of multifocal, diffuse, perihilar or unilateral GGO lacking a specific distribution or few very small GGO with a non-rounded and non-peripheral distribution) |
|  | **Atypical findings** (Absence of typical or indeterminate features AND presence of isolated lobar or segmental consolidation without GGO, discrete small nodules, lung cavitation, smooth interlobular septal thickening with pleural effusion) |
|  | **Negative for pneumonia** (No CT features of pneumonia) |

*Based on Radiological Society of North America Categorization [21]

**Table S1-5. COVID-19 severity clinical classification**

| Mild disease | Uncomplicated upper respiratory tract viral infection with symptoms such as fever, fatigue, cough (with or without sputum production), anorexia, malaise, muscle pain |
| --- | --- |
| Moderate disease | Mild pneumonia using appropriate criteria in adults (CURB-65) or children |
| Severe disease | Severe pneumonia, acute respiratory distress Syndrome (ARDS), sepsis or patients responding to noninvasive management |
| Critical disease | Respiratory failure, septic shock, and/or multiple organ dysfunctions (MOD) or failure (MOF) |

Source: Federal Ministry of Health, National Comprehensive COVID 19 Clinical Management Handbook for Ethiopia, Second Edition, September 2020 [22]
