# Supplementary material for: Attributes and factors associated with long covid in patients hospitalized for acute COVID-19: A retrospective cohort study
Source: PLoS One. 2025 Jan 16;20(1):e0317512. doi: 10.1371/journal.pone.0317512 (PMC11737794; doi:10.1371/journal.pone.0317512)
Supplement: S2 File — (DOCX) [file pone.0317512.s002.docx]

**Section 1: Socio-demographic Characteristics**

1. Age
2. Sex 0. Male 1. Female
3. BMI 0. Underweight 1. Normal weight 2. Overweight 3. Obese

**Section 2: Acute COVID-19**

1. Infection confirmed by COVID-19 PCR or RDT? 0. No 1. Yes
2. Infection status 0. Primary infection 1, Re-infection ___
3. Vaccination Status. 0. Unvaccinated 1. Vaccinated ___
4. Severity of acute COVID-19 infection. 0. Mild ___ 1. Moderate ___ 2. Severe ___ 3. Critical __
5. Symptoms during acute-COVID 19 infection (tick all that apply)

| **Symptom** | 1. **No 1. Yes** |
| --- | --- |
| Cough |  |
| Fever |  |
| Myalgias |  |
| Headache |  |
| Dyspnea (new or worsening over baseline) |  |
| Sore throat |  |
| Diarrhea |  |
| Nausea/vomiting |  |
| Anosmia or other smell abnormalities |  |
| Ageusia or other taste abnormalities |  |
| Rhinorrhea and/or nasal congestion |  |
| Chills/rigors |  |
| Fatigue |  |
| Confusion |  |
| Chest pain or pressure |  |
| Other (mention) |  |

1. Number of symptoms 1__ 2 ___ 3 ___ 4 ___ 5 or more ___
2. Duration of symptoms before admission
3. Laboratory abnormalities during acute COVID 19 infection

| **Lab Feature** | 0. No 1. Yes |
| --- | --- |
| Lymphopenia |  |
| Leukocytosis |  |
| Elevated aminotransferase levels |  |
| Elevated LDH |  |
| Elevated ESR |  |
| Elevated CRP |  |
| Elevated ferritin |  |
| Elevated D-dimer |  |
| Abnormal coagulation tests |  |
| Elevated Creatinine |  |
| Other |  |

1. Imaging abnormalities during acute COVID-19 infection

| Chest X-ray findings |  | |
| --- | --- | --- |
| Chest CT findings | Typical appearance |  |
|  | Indeterminate appearance |  |
|  | Atypical findings |  |
|  | Negative for pneumonia |  |

1. Pre-existing comorbidities (tick all that apply)

| **Comorbidities** | 0. No 1. Yes |
| --- | --- |
| Hypertension |  |
| Diabetes Mellitus |  |
| Asthma |  |
| Other chronic lung condition |  |
| HIV infection |  |
| Smoking (current and former) |  |
| Cancer |  |
| Cerebrovascular disease |  |
| Chronic liver disease |  |
| Chronic kidney disease |  |
| Heart conditions (heart failure, coronary artery disease, cardiomyopathies) |  |
| Pregnancy |  |
| Mental health disorders |  |
| Use of corticosteroids or other immunosuppressive condition |  |
| Other (state any other chronic condition) |  |

1. Date of hospitalization -
2. Date of discharge -
3. Place of admission. 0. Ward ___ 1. ICU ___
4. Length of stay in days. Ward ___ ICU ___ Total ___
5. Level of respiratory support received

| **Level of support** | **0. No 1. Yes** |
| --- | --- |
| Intranasal oxygen |  |
| Facemask oxygen |  |
| High flow nasal cannula |  |
| Non-invasive ventilation |  |
| Mechanical ventilation |  |

1. Other treatment received

| **Treatment** | 0. No 1. Yes |
| --- | --- |
| Antibiotics |  |
| Prophylactic anticoagulation |  |
| Therapeutic anticoagulation |  |
| Steroids |  |
| Anti-viral agents (Mention specific drug) |  |
| Immunomodulators (E.g. Tocilizumab) (Mention specific drug) |  |
| Other treatment received |  |

1. Complications during acute COVID-19 hospitalization

| **Complication** | 0. No 1. Yes |
| --- | --- |
| ARDS |  |
| Secondary infections |  |
| Cardiovascular complications (shock, myocardial injury, arrhythmias, heart failure) |  |
| Thromboembolic complications (VTE, stroke, limb ischemia) |  |
| Neurologic complications (encephalopathy, seizure, movement disorders) |  |
| Other |  |

**Section 3: Follow-up**

1. Duration of follow-up in days ___
2. Features on follow-up

| Follow up number | Number of days from time 0 | Symptoms reported | Laboratory abnormalities | Imaging abnormalities | Lung function test abnormalities |
| --- | --- | --- | --- | --- | --- |
| 1 |  |  |  |  |  |
| 2 |  |  |  |  |  |
| 3 |  |  |  |  |  |
| 4 |  |  |  |  |  |
