# Supplementary material for: Attributes and factors associated with long covid in patients hospitalized for acute COVID-19: A retrospective cohort study
Source: PLoS One. 2025 Jan 16;20(1):e0317512. doi: 10.1371/journal.pone.0317512 (PMC11737794; doi:10.1371/journal.pone.0317512)
Supplement: Attachment — Submitted filename: Response to Reviewers.docx [file pone.0317512.s006.docx]

**Response to reviewers**

We thank the reviewers for taking the time to assess our work again. We appreciate the suggested modifications and have revised the manuscript accordingly. The revised actions are shown in boldface type, and all revisions in the manuscript are marked using the Word “track changes” feature. Sections of the manuscript added to this letter are shown in italics.

In our previous revision, we edited our reference list and failed to state our reason why. We apologize for not clarifying why we did so. During our revision, we noted that we have mistakenly put the wrong reference on number 21 on the list. The paper we originally put on number 21 has not been retracted. However, we simply had listed the wrong paper out of human error. We have now rechecked our reference to make sure it is correct.

We have addressed each comment below.

**Reviewer**

Line 145: Is this the calculated sample size required for statistical significance? Please clarify this with a clear statement.

**Response: A statement to clarify that this minimum sample size was required for statistical significance is added.**

Line 165-167: Reference the file named S2 here.
**Response: Done**

From previous review Line 227: Other abnormalities 0.1%? What is the n value?
How many of the patients in the cohort had these “other abnormalities” that the authors are referring to?

**Response: The number of patients that had consolidation, reticulation and pleural effusion has been added as follows.**

*Additional abnormalities included pleural effusion in one patient, consolidation in two patients, and reticulations in six patients.*

For all paragraphs in the Discussion section: Authors should avoid repeating the results in the Discussion section and instead only formulate discussion points around the results that are already included in the relevant section.

**Response: We have removed sentences that were just repetitions of the results and didn’t serve a purpose to the discussion.**

Line 290-295: Are the authors still referring to the study mentioned in the previous paragraph [12] as “This study”?

**Response: The wording has been changed to “our study” to indicate we are referring to our study and not the one mentioned above.**

We would like to once again thank the reviewers for evaluating our manuscript. We would be happy to make further corrections if necessary and look forward to hearing from you soon.
